# Supplementary material for: Ultrafast motion in a third generation photomolecular motor
Source: Nat Commun. 2023 Mar 6;14:1253. doi: 10.1038/s41467-023-36777-6 (PMC9988961; doi:10.1038/s41467-023-36777-6)
Supplement: Supplementary file 1 — Supplementary Information [file 41467_2023_36777_MOESM1_ESM.pdf]

Ultrafast Motion in a Third Generation Photomolecular Motor

SUPPLEMENTARY INFORMATION

Supplementary Note 1. Materials: The compound 3GMph was available from an earlier study in which a detailed description of the synthesis and characterisation can be found.[1] Briefly, the bisketone, 2-phenyl-1H-indene-1,3(2H)-dione, was methylated using MeI with K_2CO_3 and KF on Celite and subsequently converted to the thioketone using phosphorus sulphide (P_4S_{10}) and Lawesson's reagent. A Barton-Kellogg coupling reaction with diazofluorene, followed by desulfurization of the intermediate episulfides with hexamethylphosphanetriamine, yielded the 3GMph. Solvents were spectroscopic grade (Thermo Scientific Acros) and used as received.

Supplementary Note 2. Steady-state absorption and fluorescence: Steady-state absorption spectra were recorded using a PerkinElmer Lambda XLS spectrophotometer. A 2 mm path length quartz cuvette was used and absorbance was kept below 1. Steady-state Fluorescence spectra of 3GMph were recorded in a 1 cm path length cuvette using an Edinburgh Instruments FS5 Spectrofluorimeter equipped with continuous (150 W) xenon lamp where the excitation wavelength used was 440 nm. Supplementary Figure 1 shows absorption and fluorescence spectra of 3GMph in acetonitrile (ACN) and cyclohexane (CHX).

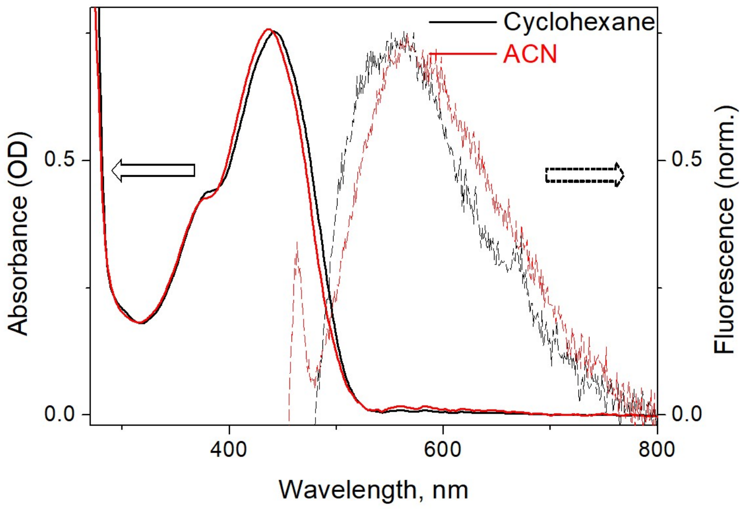

Supplementary Figure 1: Steady-state absorption and emission spectra of 3GMph in both CHX and ACN. Both the spectra are slightly solvent dependent, with an increased Stokes shift in ACN.

Supplementary Note 3. Emission quantum yield measurements: The emission quantum yield (Q) of the motor (3GMph) in CHX and ACN were determined. FAD in tris pH-8 buffer was used as reference standard with a quantum yield of 0.03.[2] Concentrations of all the samples were kept between 0.1-0.2 OD in a 1 cm cell; absorption spectra are shown in Supplementary Figure 2a. The emission spectra for sample and reference were collected (Supplementary Figure 2 b,c,d) keeping all the parameters (data accumulation time, slit width) the same. Yields were calculated according to:

$$Q_f = Q_r \cdot \left(\frac{I_f}{I_r}\right) \cdot \left(\frac{A_r}{A_f}\right) \cdot \left(\frac{n_f^2}{n_r^2}\right)$$

Where Q_f = Quantum yield of 3GMph fluorescence; Q_r= quantum yield of FAD reference; A= absorbance (at 440 nm); *I* = integrated emission area; *n* = refractive index. The recovered quantum yields were 0.9×10^{-5} (CHX) and 1.6×10^{-5} (ACN). However, a significant error must be associated with the measurement due to very weak emission (see Supplementary Figure 2 c,d), so we only conclude in both cases the yield to be $<3 \times 10^{-5}$.

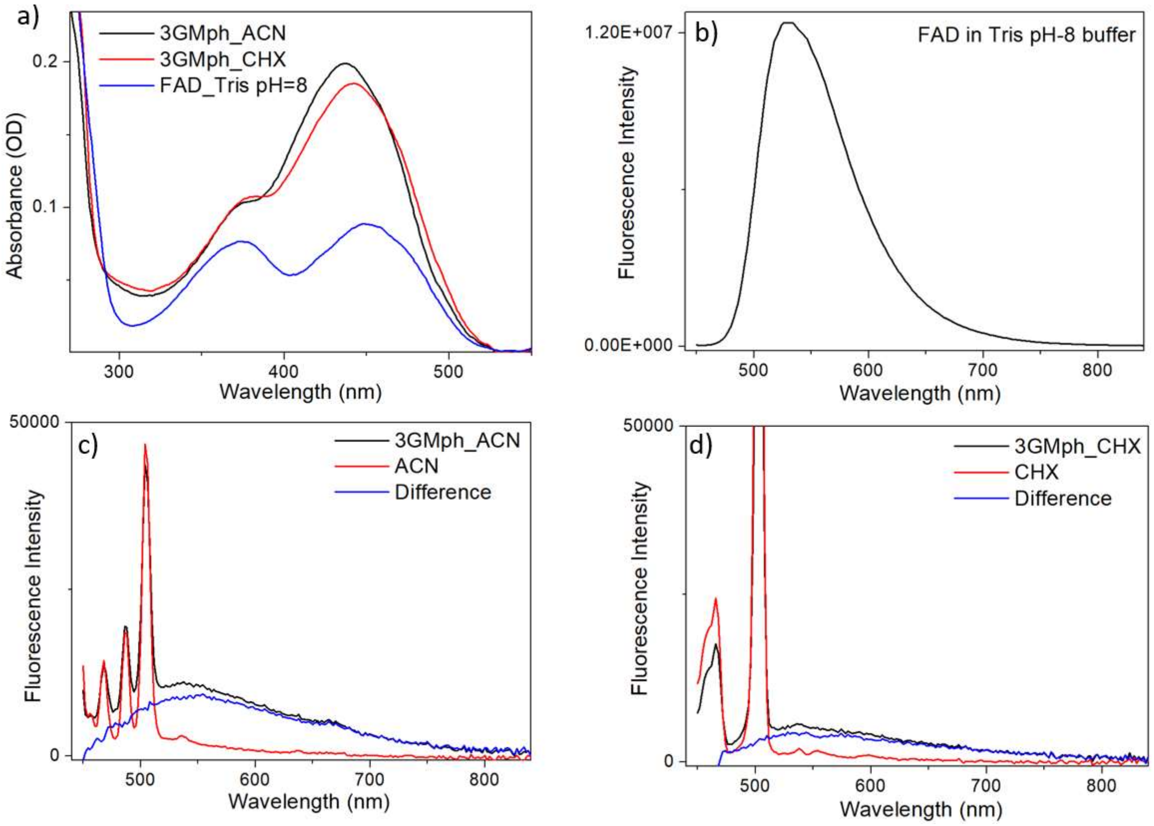

Supplementary Figure 2: a) Raw steady-state absorption spectra of 3GMph (in both CHX and ACN) and FAD (in Tris pH-8 buffer) in 1 OD cell. b-d) Corresponding raw emission spectra are plotted for FAD and 3GMph in ACN and CHX respectively. Emission from 3GMph is very weak and has contributions from solvent Raman and instrument artifact (red). The difference plot (blue) shows the recovered emission spectra from 3GMph, which was used for quantum yield determination of 3GMph (Supplementary Note 3).

Supplementary Note 4. Time-resolved Fluorescence Up-Conversion (TRUC): The detailed description of the TRUC set up was reported elsewhere.[3] Briefly, the femtosecond oscillator produced pulses centred at 820 nm with a time duration of around 20 fs (after compression) at a 76 MHz repetition rate and a power of 920 mW. The laser output was passed through a fused silica prism compressor and then focused onto a type I BBO crystal (50 μm thick) with a 150 mm focal length concave mirror to produce up to 15 mW of 410 nm second harmonic excitation pump beam. The pump (second harmonic) and fundamental (which was used as the Gate pulse) beams were separated with a dichroic mirror. The gate beam was compensated for dispersion (which is introduced by the

dichroic mirror and the focusing lens) using a pair of chirped mirrors (Femtolasers GSM216) and is directed through an optical delay line (Physik Instrumente). The pump beam passes through another pair of chirped mirrors (Femtolasers GSM012) to compensate for the dispersion introduced by the sample cell window. The 410 nm pump beam was focused at the sample cell (1 mm path length) with a 150 mm concave mirror. The fluorescence generated from the sample was focused onto a BBO crystal using a 15 times magnification reflective microscope objective with a back focal length of 160 mm. Finally, the fluorescence and gate (820 nm) beams were focused and mixed in a type I BBO crystal (100 μm). Intensity of the resultant up-converted light (at wavelengths from 307 to 353 nm depending on the fluorescence wavelength measured) were detected by a photomultiplier and monochromator combination and measured with a photon counter. The intense scattering from the pump beam was attenuated by a filter (GG455, Schott) placed after the objective (before the up-converting BBO crystal) and another filter (UG11, Schott) at the entrance slit of the monochromator isolated the up-converted signal. The IRF and time zero for the experiment was determined to be around 43 fs by recording the upconversion of Raman scattering from heptane at 470 nm. All reflective optics were used to minimise dispersion and pulse broadening. All TRUC measurements were carried out with a flowing solution driven by a peristaltic pump. Photo-damage was minimised using a flow rate suitably adjusted 0.5 mL s^{-1} and pump pulse energy of 5 mW (focal spot of ca 200 μm inside cuvette). The fast recovery time from the metastable to stable form as well as sufficient volume (150 mL) of the solution ensured that build-up of the metastable form was negligible. The analyses of TRUC measurements were carried out using IGOR pro 5 wavemetrics software that yields decay time constants and their respective amplitudes.

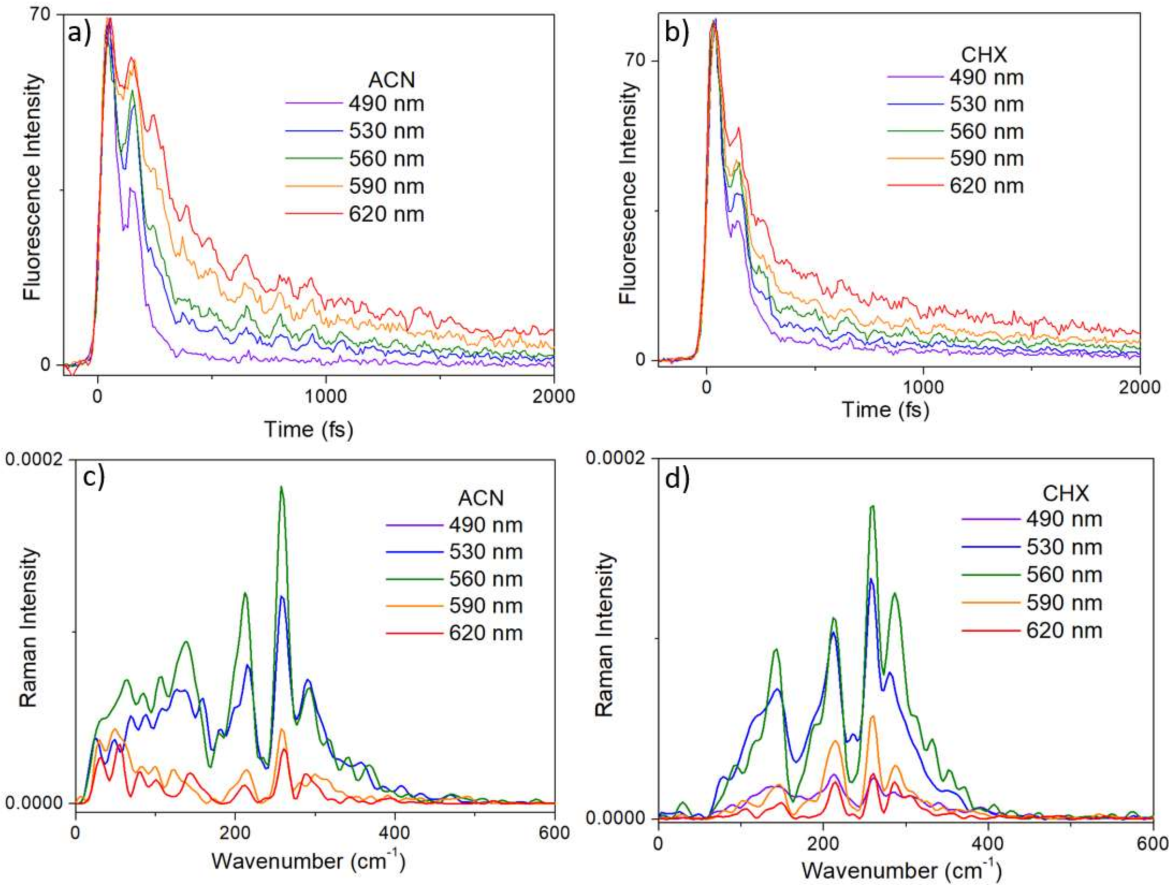

Supplementary Figure 3: Time-resolved fluorescence dynamics at different emission wavelengths in a) ACN and b) CHX. Both show non-single-exponential relaxation and wavepacket modulation. These data can be well fitted by a biexponential decay function giving sub- ps and few-ps timescale decay constants (Supplementary Table 1). Relative contribution of the few-ps component increases from 490 to 620 nm. The residual from the fitting isolates the oscillatory wavepacket dynamics (see below). Fourier transformation of these are shown in c) ACN and d) CHX displaying the corresponding frequencies. The four modes: 146, 213, 259, and 290 cm^{-1} are present in both the solvents. However, the additional 50-60 cm^{-1} mode is consistently present only in ACN. This indicates the role of solvent polarity on this mode.

ACN Emission wavelength /nm	τ_1 /fs	a_1	τ_2 /ps	a_2
490 nm	103±12	0.99	2.42±1.2	0.01
530 nm	155±15	0.94	1.95±1.5	0.06
560 nm	178±20	0.89	1.59±0.43	0.11
590 nm	220±25	0.82	1.6±0.5	0.18
620 nm	292±15	0.79	2.3±1	0.21

CHX Emission wavelength /nm	τ_1 /fs	a_1	τ_2 /ps	a_2
490 nm	87±10	0.95	1.21±0.61	0.05
530 nm	111±10	0.92	1.32±0.4	0.08
560 nm	125±10	0.88	1.52±0.37	0.12
590 nm	130±9	0.84	1.56±0.22	0.16
620 nm	143±11	0.76	1.63±0.17	0.24

Supplementary Table 1. Fitting parameter (a_i are pre-exponential weights) of the TRUC data of 3GMph in ACN and CHX.

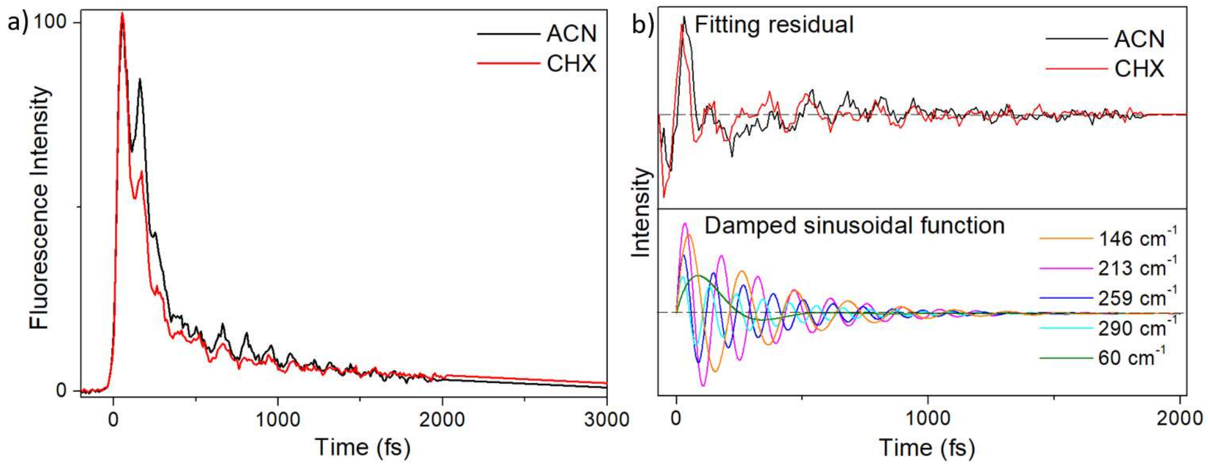

Supplementary Figure 4: a) Time-resolved fluorescence kinetics (at 560 nm) of 3GMph in ACN and CHX are plotted together. b) Biexponential fitting of the kinetics provides the corresponding fitting residuals, which were analysed by FT in Supplementary Figure 3. The oscillations in the residuals are solvent polarity dependent, and here were fit in the time domain. The individual damped sinusoidal oscillations corresponding to the fitting of wavepacket modulation (Supplementary Figure 5) are shown. The 60 cm^{-1} contribution is only detected in ACN, consistent with the FT analysis.

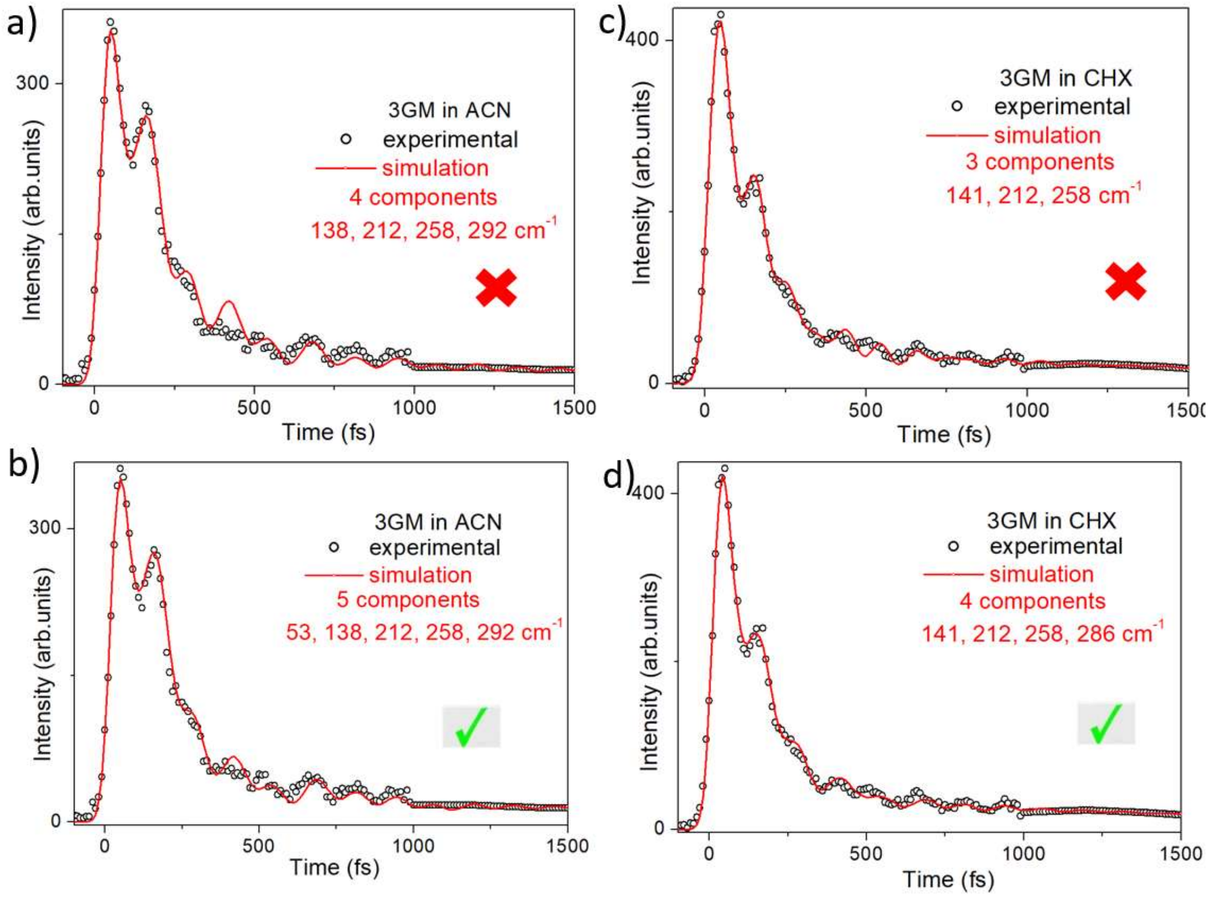

Supplementary Figure 5: Fitting of the upconversion datasets using a decay function comprised of the sum of two exponential decay terms and a-b) four or five damped harmonic oscillators in ACN and c-d) three or four damped harmonic oscillators in CHX. Five component fitting is required in ACN and only four component fitting in CHX, again in good agreement with the earlier analyses. Thus, the additional component in ACN is required to better fit the raw data consistent with time and frequency domain analyses.

Supplementary Note 5. Femtosecond transient absorption measurements (TA): The detailed description of the transient absorption set up used here has been presented elsewhere.[4] In brief, TA is a two pulse technique – a femtosecond pulse is the pump and a time delayed broadband white light continuum is the probe. The fundamental beam from the Spectra Physics Mai Tai laser oscillator is amplified in a Ti:sapphire regenerative amplifier (Spectra Physics Spitfire ACE) to generate output pulses centered at 800 nm with duration of 120 fs, repetition rate of 1 kHz and energy of 5 mJ per pulse. This amplified pulse output drives two commercial optical parametric amplifiers (OPA, Light Conversion TOPAS Prime). One OPA is used to generate the 440 nm pump pulse with duration of 80 fs, which electronically excites the samples. The pump pulse was passed through a mechanical chopper (500 Hz), a computer-controlled delay stage and a half wave plate and polarizer (to set polarization at magic angle). The second OPA is tuned to generate pulses at 1250 nm which was attenuated (10 μJ) and then focused on to a 3 mm thick sapphire window to generate broadband white light continuum (500-1400 nm). To access the 400-800 nm spectral window, we directly focused the fundamental 800 nm beam onto the sapphire plate to generate the WLC probe. Spectra were measured in two separate overlapping WLC windows and then stitched together.

The WLC before the sample was split by a 50/50 beam splitter. One part (Reference) was used in the reference detection channel to correct for intensity fluctuations in the probe spectrum. The second part (Probe) was spatially overlapped with the pump pulse in a 1 mm thick sample cell. Both the pump and probe pulses were focused, with focal spot sizes of 270 μm and 50 μm respectively. The sample was circulated through the 1 mm sample cell which had 0.5 mm thick fused silica windows. Pump beam energy was attenuated to 0.25 mW at the sample cell in order to avoid any photodamage. After passing through the sample the probe was aligned collinearly with the reference beam and on top of each other and both were dispersed using a home-built prism based spectrograph. Both the dispersed beams were then focused and detected by two separate synchronised 16 bit A/D CCD detectors from Entwicklungsbüro Stresing (1024 pixels). A chopper at 500Hz in the pump beam path allowed the detection of pump-on/pump-off probe and reference spectra. The referenced difference spectrum for each pulse pair was calculated using:

$$\Delta A = -log(\frac{Probe\ (Pump\ on)\ \mathrm{X}\ Refernce\ (Pump\ off)}{Reference\ (Pump\ on)\ \mathrm{X}\ Probe\ (Pump\ off)})$$

The detector was calibrated using Mercury-Argon lamp (HG-1 Ocean Optics). The instrument response function, IRF, with 440 nm excitation pump and probe was determined to be about 100 fs as obtained from the solvent Kerr response. The data presented were average over 7 cycles and each time trace was accumulated for 0.1 sec. All pump-probe measurements were done in 1 mm cell under flowing condition with the liquid driven by a peristaltic pump.

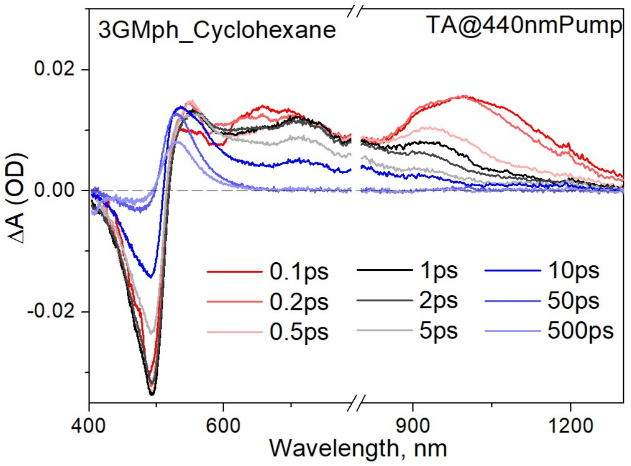

Supplementary Figure 6: Transient absorption spectra of 3GMph in CHX recorded at different pump-probe delays. Excitation pump used was at 440 nm.

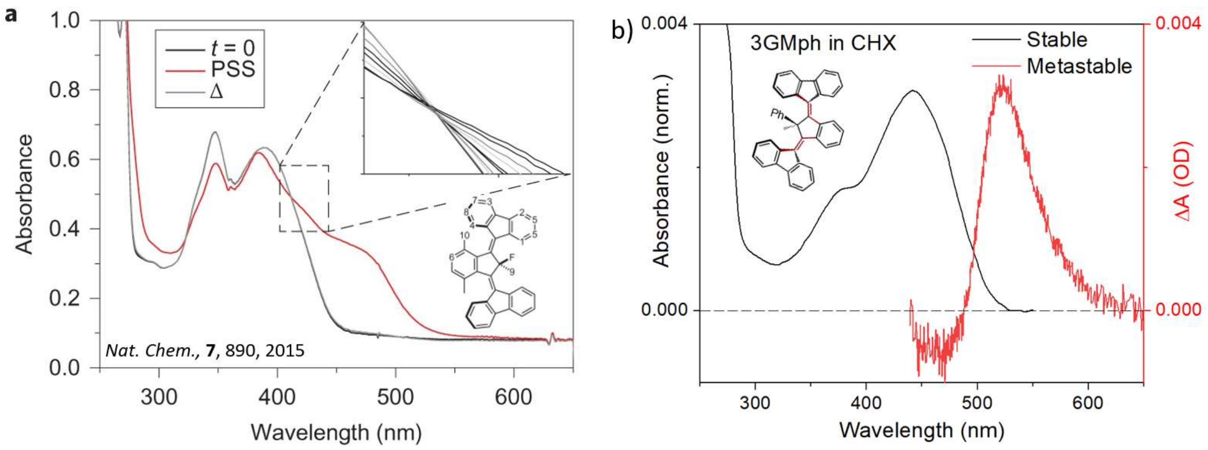

Supplementary Figure 7: a) Reported absorption spectra of the stable (*t* = 0 s, black) and photostationary state (PSS, red) of 3GM-F (previously published by the authors in Nat. Chem. 7, 890, 2015). The new absorption feature at 480 nm corresponds to the metastable form and is red-shifted compared to the stable form absorption. b) Steady-state absorption spectrum (black) and transient absorption difference spectrum (at 1ns) probe stable and metastable form of 3GMph respectively. The metastable form absorption contributes at 530 nm and the spectrum is red-shifted compared to the stable form absorption, consistent with the example in a).

Supplementary Note 6. Global Analysis: We used Global analysis for accurate assignment of the transient species and recovery of their respective lifetimes and Evolution Associated Difference Spectra (EADS). The TA dataset were analyzed using the Glotaran software package, which reports both EADS and lifetimes associated with each species.[5] Here we used three states connected in a sequential scheme: bright Franck-Condon state → dark state → product. In Supplementary Figure 8, we show evolution associated decay spectra for 3GMph in both solvents. It shows three time components corresponding to bright state (sub-ps), dark state (few-ps) and metastable product (ns). The dark state lifetime is sensitive to solvent polarity. The good quality of fit has been verified by looking at the wavelength dependent kinetic fittings (see main Figure 2d) and associated residuals. Steady state spectra (black dotted lines) help to assign bleach and stimulated emission.

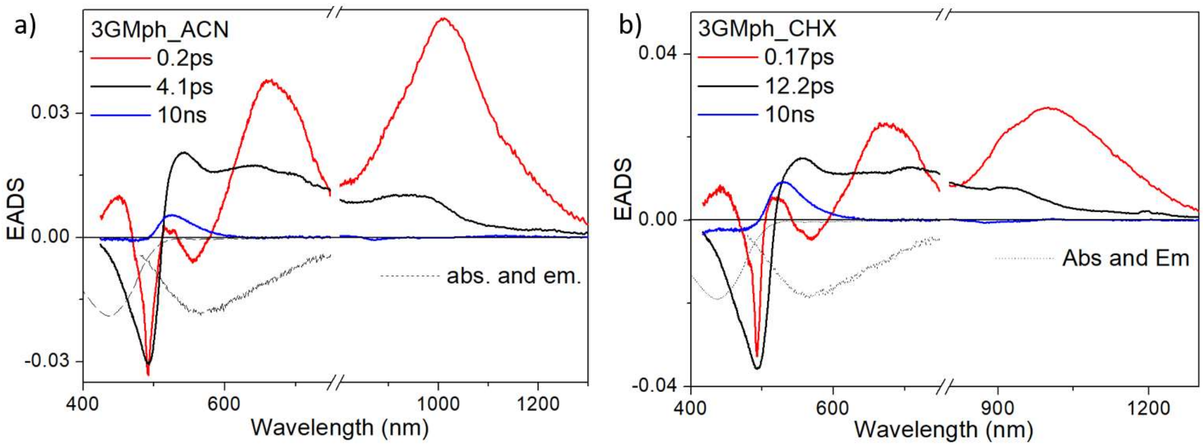

Supplementary Figure 8: Global analysis of the transient absorption data of 3GMph in a) ACN and b) CHX. Three component sequential model was the simplest model to accurately fit the TA spectral kinetics, as is shown in main Figure 2d. The components are 0.2 ps, 4.1 ps and 10 ns in ACN while these are 0.17 ps, 12.2 ps and 10 ns in CHX. These can be assigned to bright state, dark state and metastable product spectra respectively. The lifetime of the dark state component is reduced by 3 times in polar ACN. Absorption and emission spectra (grey dotted lines) are plotted to distinguish ground state bleach and stimulated emission features.

Supplementary Note 7. Femtosecond stimulated Raman measurements (FSRS): The setup used for TA measurement was modified for the FSRS measurements. FSRS is a three-pulse technique: the femtosecond 'actinic' pump excites the stable ground state molecule. The sample is probed by a narrowband (10 cm^{-1}) picosecond Raman pulse in the presence of a broadband femtosecond probe pulse, which together stimulate the coherent Raman scattering process from the sample. The white-light continuum probe (generated using 1250 nm OPA output) and actinic pump pulses were generated as described in the TA (Supplementary Note 5). The Raman pulse was generated by sending part of the fundamental amplified beam (from Spectra Physics Spitfire ACE amplifier) through a commercial second harmonic bandwidth compressor (SHBC, Light Conversion) and then a picosecond configured OPA (Light Conversion, TOPAS-PS). The Raman pulse was tuned (560 and 650 nm) to be in resonance with the excited state absorption of 3GMph. Raman pump at 650 nm is in resonance with the bright and dark state while that at 560 nm the resonance with the bright state, dark state and metastable product. Both are resonant with the very broad emission, though 650 nm much less so. The spectral resolution for FSRS was determined by the pulse-width of the Raman pump which was set at about 2 ps. All the three beams- Actinic pump, Raman pump and white–light probe pulse are focused (spot sizes 150, 250 and 50 μm respectively) and overlapped spatially and temporally inside the flow cell. No reference Probe was used for FSRS. Stimulated Raman from cyclohexane was used to optimize temporal overlap between Probe and Raman pump. Delay between actinic pump and Raman probe is controlled by a computer-controlled delay stage. The transmitted probe beam was dispersed in a high spectral resolution (<10 cm^{-1}) grating spectrometer (SPEX 500M) and the probe was detected using a single CCD (1024 pixel). The detector was calibrated using Raman peaks of cyclohexane (reference sample). Each spectrum was accumulated for 6 s and a total of 10 scans were recorded using LabView controlled software. The actinic pump power used was 1 mW at 440nm for all four samples. Raman pump was set at 560nm (4 mW) and 650nm (4mW). All the FSRS measurements of 3GMph were done in both polar acetonitrile and nonpolar cyclohexane under similar experimental conditions (overlap, power, concentration) so that we can compare the effect of solvent polarity. The sample absorbance (at 440nm) was kept at 0.7 OD during all measurements. Large volumes (150ml) of sample were used and

circulated continuously through a 1 mm flow cuvette with the liquid driven by a peristaltic pump to replenish molecule for each pump pulse. The sample integrity of the solution is checked by recording ground state absorption before and after FSRS measurement. Time resolution of the experiment is dictated by the actinic pump and probe pulse convolution which is around 100 fs, as obtained from solvent response in methanol.

Spectra were recorded at 1 kHz. Two synchronized choppers operating at 250 Hz and 500 Hz were used to modulate the Raman pump and actinic pulses respectively. This result in four different signals: i) Excited state FSRS (Probe + Raman + Actinic); ii) Transient absorption (Probe + Actinic); iii) Ground state FSRS (Probe + Raman) and iv) Probe reference (probe only). The unprocessed FSRS signal is obtained when Actinic pump, Raman pump and probe pulses are present on the sample as we detect log ($I_{Raman+Actinic+Probe}/I_{Probe}$). Along with excited state Raman, this unprocessed FSRS signal also contains information on ground state Raman (log ($I_{Raman+Probe}/I_{Probe}$)), transient absorption (TA) (log ($I_{Actinic+Probe}/I_{Probe}$)) and nonlinear background.

Processing of the unprocessed FSRS signal at 200 fs has been shown in the Supplementary Figure 23. The unprocessed FSRS signal (grey solid line) is obtained when actinic pump, Raman pump and probe pulses are present on the sample as we detect log ($I_{Raman+Actinic+Probe}/I_{Probe}$). On the other hand, the actinic pump and probe pulses (i.e. Raman pump OFF) generate transient absorption signal (log ($I_{Actinic+Probe}/I_{Probe}$) as blue dotted line) while the Raman pump and probe pulses (i.e. actinic OFF) provide ground state FSRS signal (log ($I_{Raman+Probe}/I_{Probe}$) as black solid line). In our experiment, the ground state Raman signal has both solvent (large signal marked by asterisk) and motor contributions (numbered frequency). The unprocessed FSRS signal contains information on the excited state Raman along with the ground state Raman, transient absorption (TA) and nonlinear background. Therefore the ground state Raman and TA contributions have been subtracted from the unprocessed FSRS data. The resultant difference spectra (say, raw excited state Raman signal (ESR_{lambda})) are shown as red solid line. Same procedure is followed to produce raw ESR data at different time delays which are shown in Supplementary Figures 9a and 10a. These are baseline corrected to obtain the raw excited state Raman

signal as shown in Supplementary Figure 9b and 10b. This same procedure is followed for different pump-probe time delays. The FSRS signal processing to obtain the bleach filled ESR (main Figure 3c) as described in Supplementary Note 9.

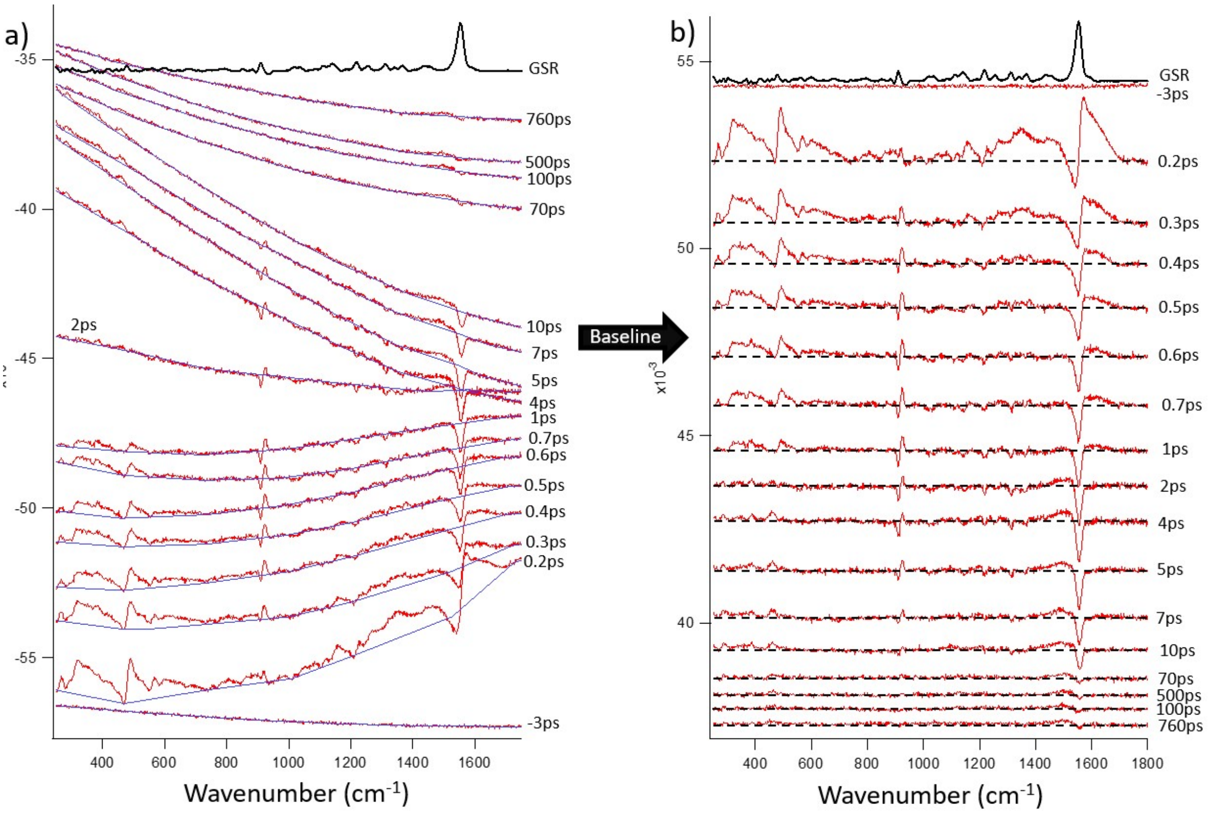

Supplementary Figure 9: a) Raw excited state Raman data (ESR, Raman pump at 560 nm) of 3GMph in ACN at different time delays with the corresponding baselines shown. The ground state Raman (GSR, black spectrum) is plotted for comparison. b) The baseline corrected raw ESR spectra are shown (note the large negative contributions at the ground state wavenumber).

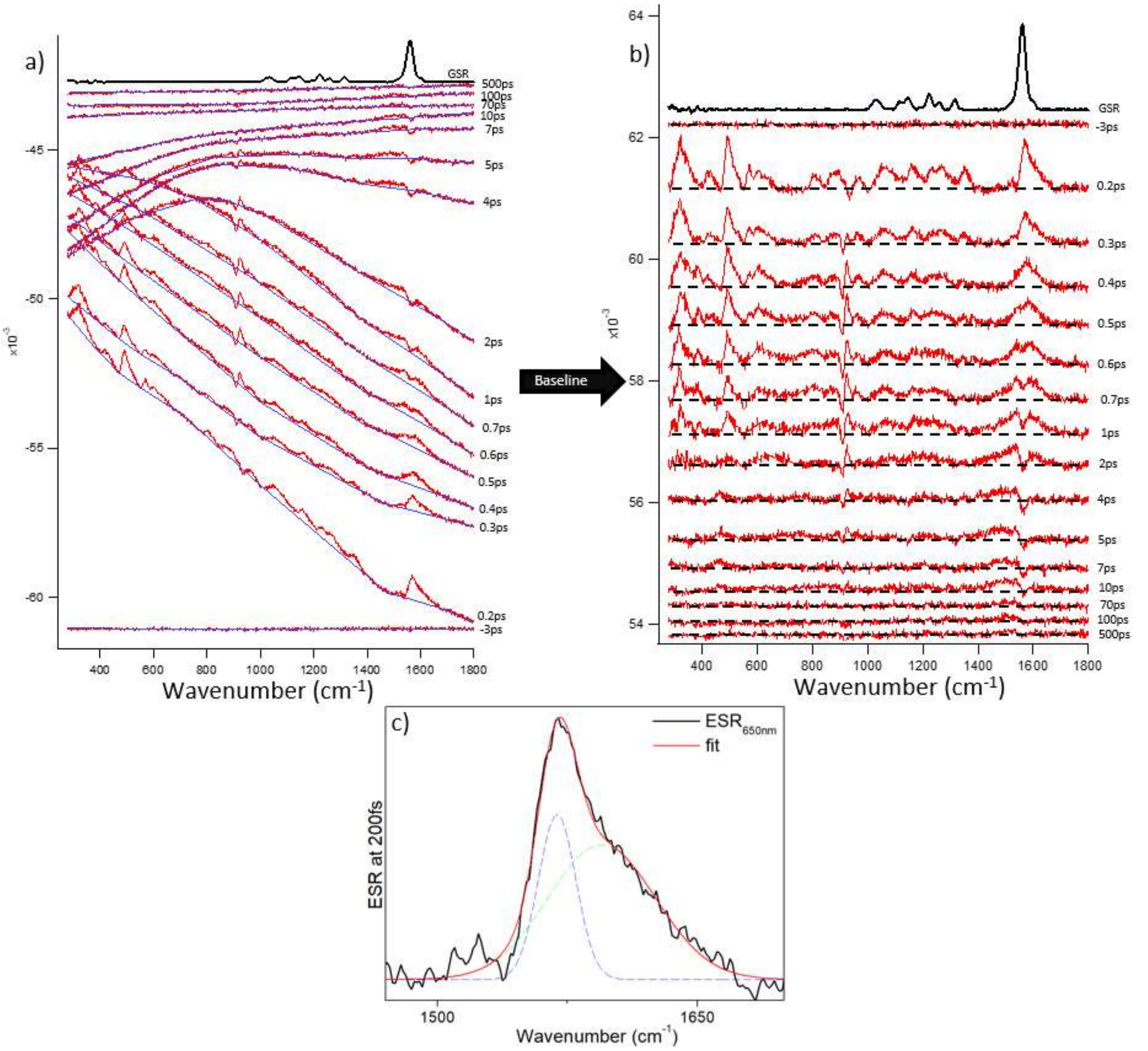

Supplementary Figure 10: a) Raw excited state Raman data (ESR, Raman pump at 650 nm) of 3GMph in ACN at different time delays with the corresponding baselines are shown. The ground state Raman (GSR, black spectrum) is plotted for comparison. b) The baseline corrected raw ESR spectra are shown. c) Peak fittings of the raw ESR spectra at 200 fs in axle mode region. Two Gaussian functions were used to fit the spectrum (blue and green dotted spectra with peaks at 1565 and 1600 cm^{-1} respectively). Note the greatly reduced negative component in b).

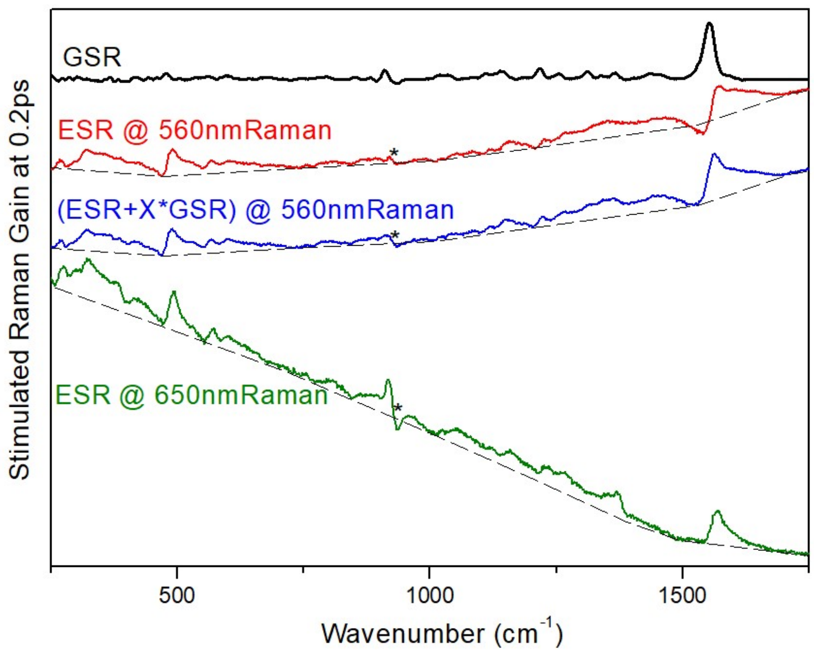

Supplementary Figure 11: Procedure for baseline correction and bleach filling of the excited state Raman (ESR) data in main Figure 3a is presented here. The ground state Raman (GSR) spectrum is obtained after solvent subtraction from the FSRS data (measured with no actinic pump) followed by baseline correction. The red spectrum represents raw ESR_{560nm} data (Raman pump at 560 nm) collected at 200 fs time delay. The baseline for this ESR data has been drawn. Similarly, baselines for the raw ESR_{650nm} data (green spectrum; Raman pump @ 650 nm) and bleach filled ESR_{560nm} data have been drawn. Asterisk (*) shows the region of solvent artifact.

Supplementary Note 8. Spectral Curve Fitting: The Gaussian peak fittings (for Figure 3d and Supplementary Figure 10c, 14d) were performed using OriginPro software varying the peak widths and peak frequencies. This provides good quality fits in the 1520-1730 cm^{-1} region for all datasets from 200-400 fs timescale using two Gaussian functions. They show two peak frequencies at around 1565 and 1607 cm^{-1} as shown in Supplementary Table 2.

Time/fs	Peak/cm^{-1}	Width /cm^{-1}	Amplitude
200fs	1565	23	0.053
	1607	75	0.11
300fs	1562	24	0.03
	1604	76	0.06
400fs	1560	27	0.02
	1600	77	0.037

Supplementary Table 2. Fitting parameters of the FSRS C=C stretch Gaussians fir to bright state data in main Figure 3d.

Supplementary Note 9. FSRS Bleach filling procedure: The raw FSRS signal (Supplementary Figure 9a and 10a) of 3GMph contain negative contributions. The behavior of this spectrum with wavelength (see main text) suggests the origin is the bleached near resonant ground state. To correct for this the raw excited state Raman signal is bleach filled by adding ground state Raman (GSR) of 3GMph as follows:

$$ESR = ESR_{RAW} + (X * GSR)$$

where, X is the amount of GSR to be added. We started the procedure with ESR_{RAW} signal measured at 760 ps where there is a positive contribution from only the metastable product (see black spectrum in Supplementary Figure 12a). The value of X was varied to fill the observed bleach until we arrive at the physically realistic spectrum (*i.e.* when there is neither bleach nor extra addition in the negative region). The ESR spectrum (at 760 ps) with X = 0.2 is selected. The rest of the time traces were then filled with the value of X fixed following the ground state bleach recovery dynamics measured from TA (Supplementary Figure 12b). Thus, X is not arbitrary but reflects the amount of bleaching. The bleach filled spectra are shown in main Figure 3c and Supplementary Figure 13b, 14b, 15b for all four measurements (two solvents, two Raman pump wavelengths).

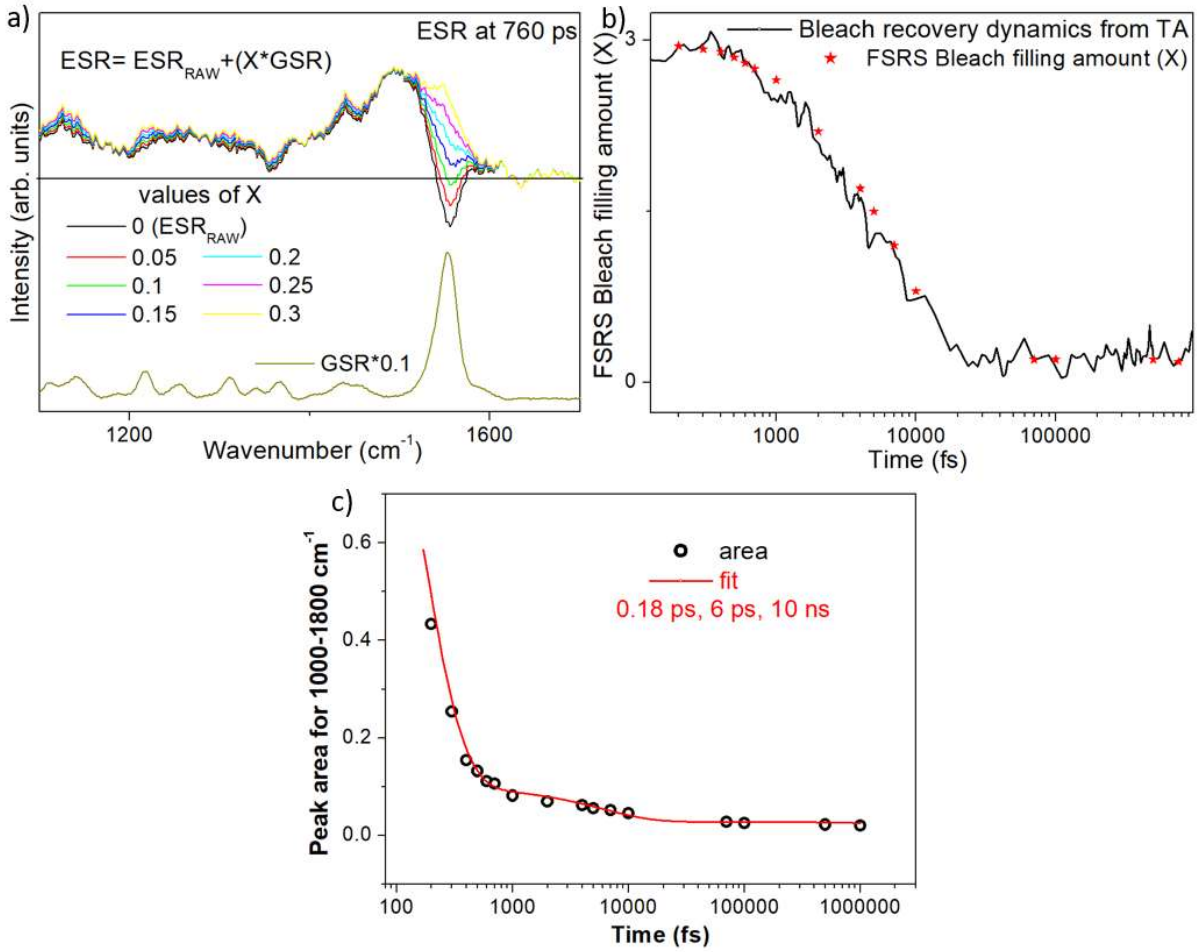

Supplementary Figure 12: Bleach filling of ESR data of 3GMph in ACN with Raman pump at 560 nm. a) Different amounts (X) of GSR spectrum have been added to the raw ESR data at 760 ps (black spectrum, ESR_{RAW}). The bleach filled ESR spectrum keeping X = 0.2 at 760 ps yields a single spectral profile. b) The rest of the time points are bleach filled so that the bleach filling amount (X) at different times follows ground state recovery dynamics from TA. c) Peak area dynamics for the region 1000-1700 cm^{-1}. It is fitted by decay times of 0.18 ps, 6 ps, and 10 ns.

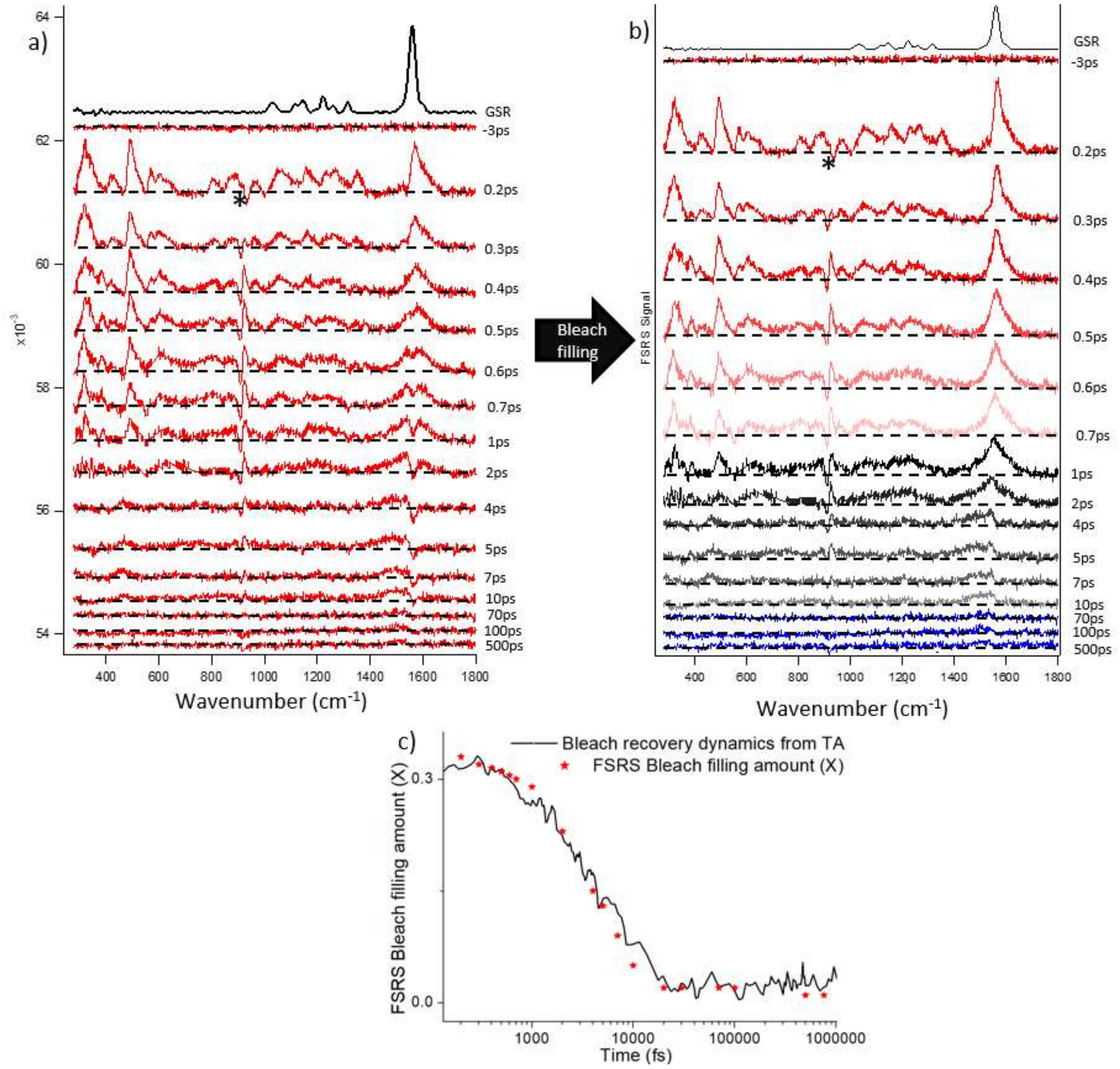

Supplementary Figure 13: Bleach filling of ESR data of 3GMph in ACN with Raman pump at 650 nm. a) Baseline corrected raw ESR spectra and b) the bleach filled ESR spectra at different time delays are shown. Asterisk (*) shows the region of solvent artifact. c) The corresponding bleach filling amount at different time delays are plotted (marked by red star). The bleach filling dynamics from FSRS follows the ground state bleach recovery dynamics from TA (black line).

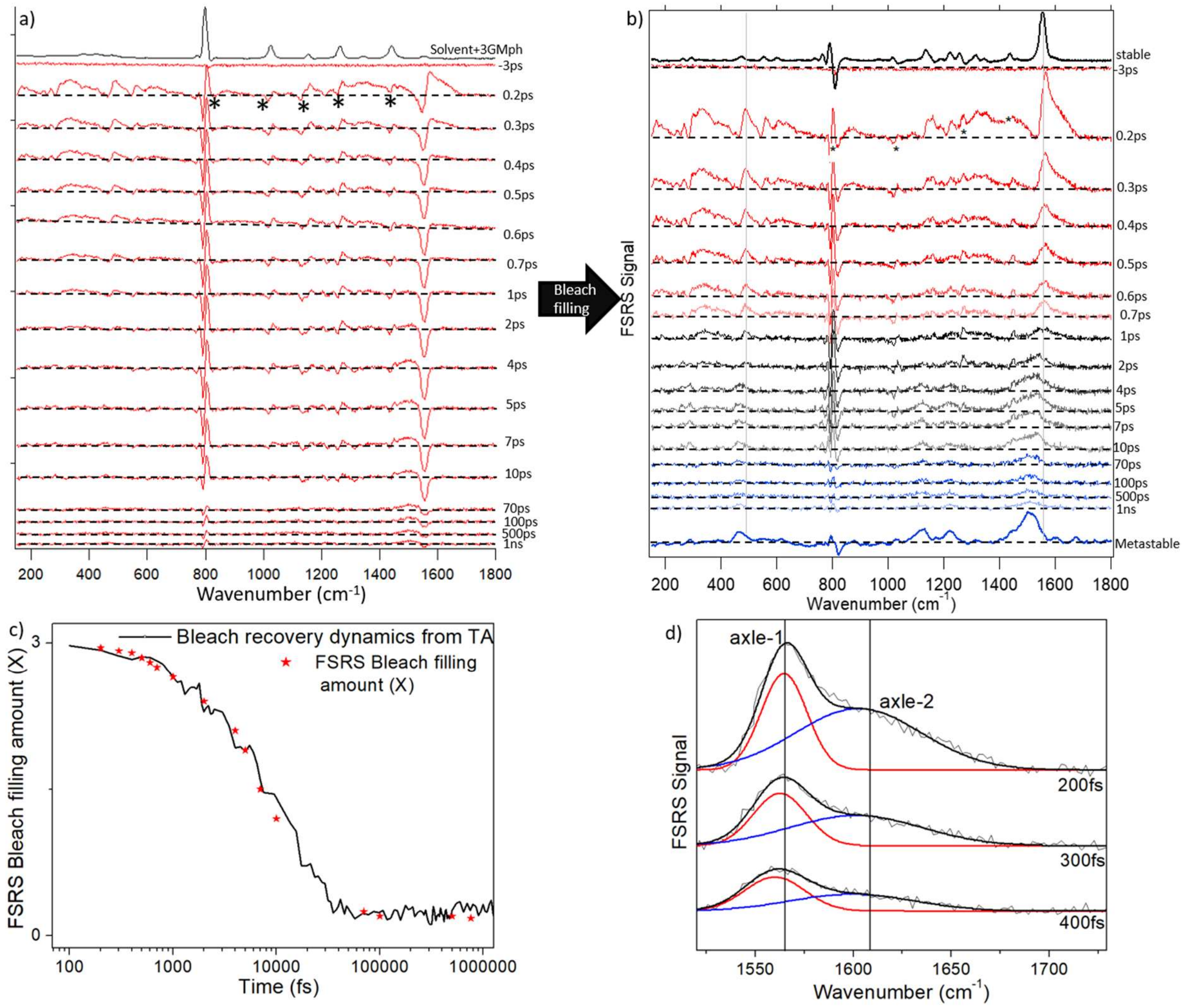

Supplementary Figure 14: Bleach filling of ESR data of 3GMph in CHX with Raman pump at 560 nm. a) Baseline corrected raw ESR spectra and b) the bleach filled ESR spectra at different time delays are shown. The ground state Raman of stable and metastable (top and bottom spectra) are plotted for comparison. Asterisk (*) shows the region of solvent artifact. c) The corresponding bleach filling amount at different time delays are plotted (marked by red star). The bleach filling dynamics from FSRS follows the ground state bleach recovery dynamics from TA (black line). d) Peak fittings of the axle mode region in the initial timescales are shown. Two Gaussian functions were used to fit the spectrum and can be represented by axle-1 and axle-2.

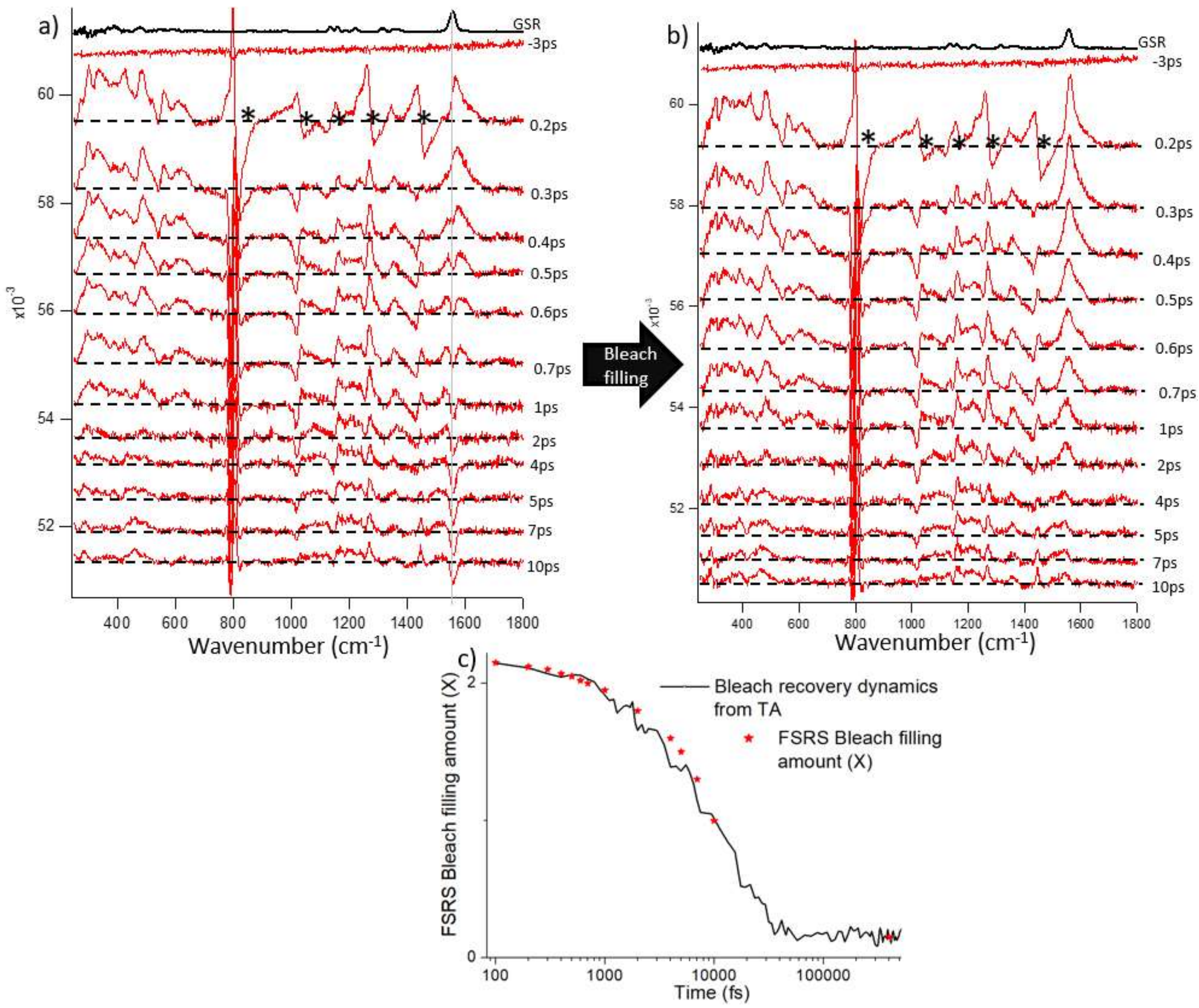

Supplementary Figure 15: Bleach filling of ESR data of 3GMph in CHX with Raman pump at 650 nm. a) Baseline corrected raw ESR spectra and b) the bleach filled ESR spectra at different time delays are shown. Asterisk (*) represents the solvent artifacts. c) The corresponding bleach filling amount at different time delays are plotted (marked by red star). The bleach filling dynamics from FSRS follows the ground state bleach recovery dynamics from TA (black line). Baseline corrected a) raw ESR data and b) bleach filled ESR data of 3GMph in CHX with Raman pump 650 nm.

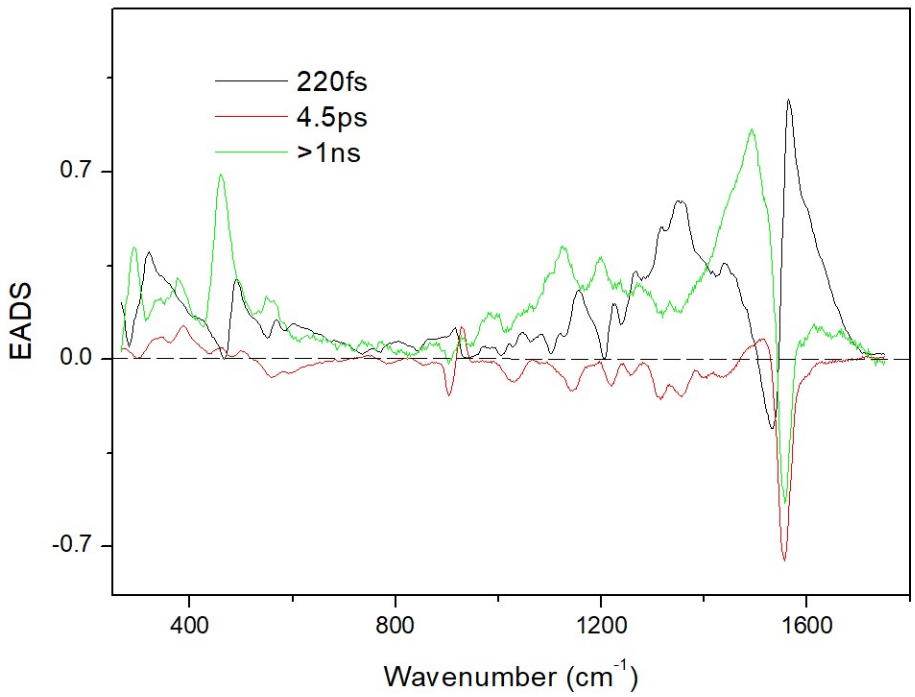

Supplementary Figure 16: Evolution associated difference spectra of the baseline corrected raw ESR data (Supplementary Figure 9b) of 3GMph in ACN with Raman pump at 560nm. This provides the similar time-components as was obtained with the bleach filled ESR global analysis (Figure 3e). Thus the bleach filling has not perturbed the recovered kinetics.

Supplementary Note 10. Computational details: All calculations were carried out using Gaussian16.[6] Molecular geometry optimization and non-resonant Raman spectra calculations for the stable and metastable form of 3GMph were carried out in gas phase conditions within Density Functional Theory at the rb3lyp/tzvp level. Both the forms were first optimized for structure and then Raman spectra were calculated. All frequencies have been scaled by a factor of 0.98 (to account for a systematic error in the DFT predictions) and then plotted as shown by red vertical lines in Supplementary Figure 17. The experimental GSR spectra are plotted (grey spectra) for comparison which shows quite good matching between calculated and experimental spectra, although low frequency modes are enhanced in the metastable experimental spectrum.

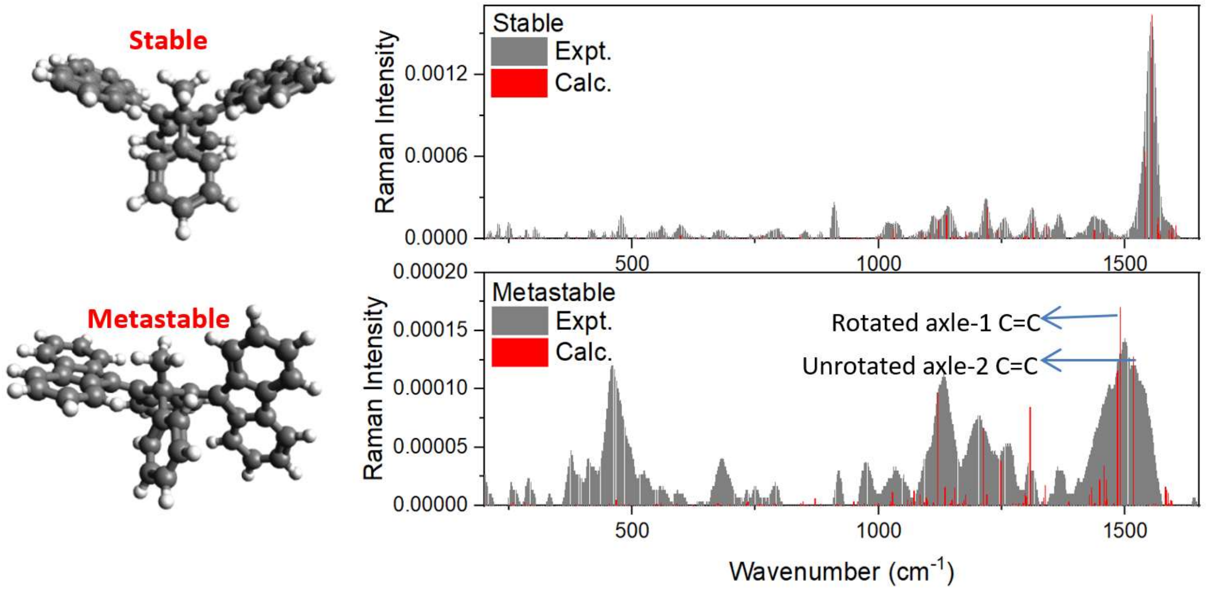

Supplementary Figure 17: DFT calculation for structure and non-resonant Raman spectra (red vertical lines) of stable and metastable forms. The calculated spectra are plotted on top of the experimental spectra (Grey). The high-frequency regions of the calculated and experimental data are in good agreement with each other. The stable form shows symmetric and anti-symmetric C=C stretching (of both the axles 1 and 2) at 1540 and 1554 cm^{-1} respectively. On the other hand, the metastable form shows two distinct C=C stretching frequencies (at 1490 and 1516 cm^{-1}) for the two axles: axle-1 (rotated) and axle-2 that (not rotated). The low-frequency 480 cm^{-1} mode in the calculated spectra of metastable form is weaker than the experimental data probably due to experimental data being collected under pre-resonant condition.

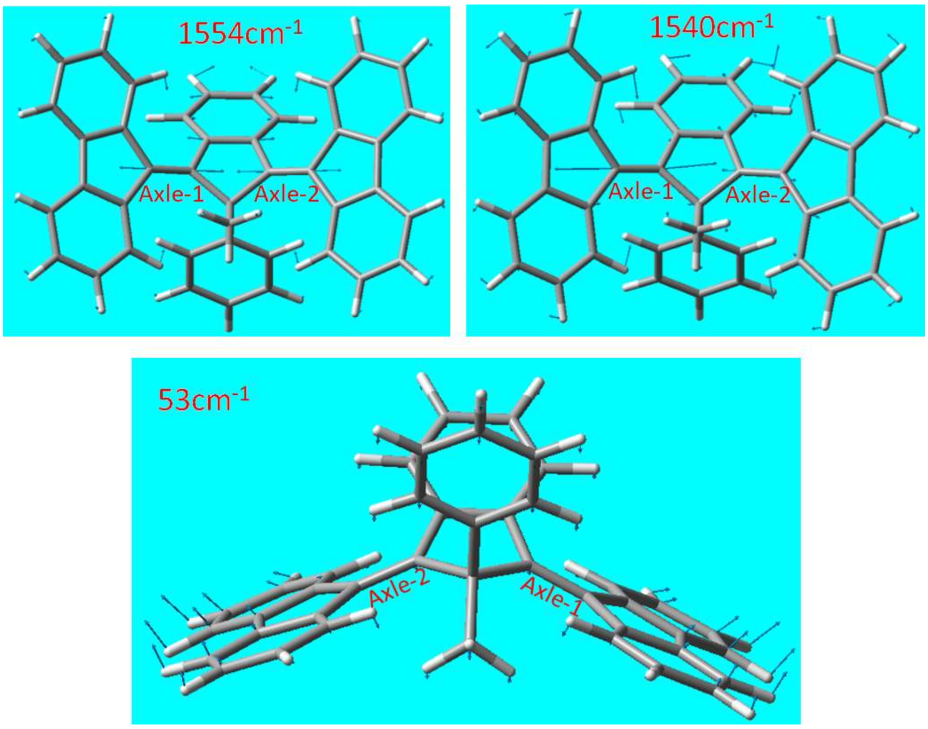

Supplementary Figure 18: 1554, 1540 and 53cm^{-1} modes obtained from Gaussian DFT calculation of the stable form. The 1554 and 1540 cm^{-1} modes represent symmetric and anti-symmetric ethylenic C=C stretching of the two axles respectively. The 53 cm^{-1} mode represents a flapping-like motion of two rotors.

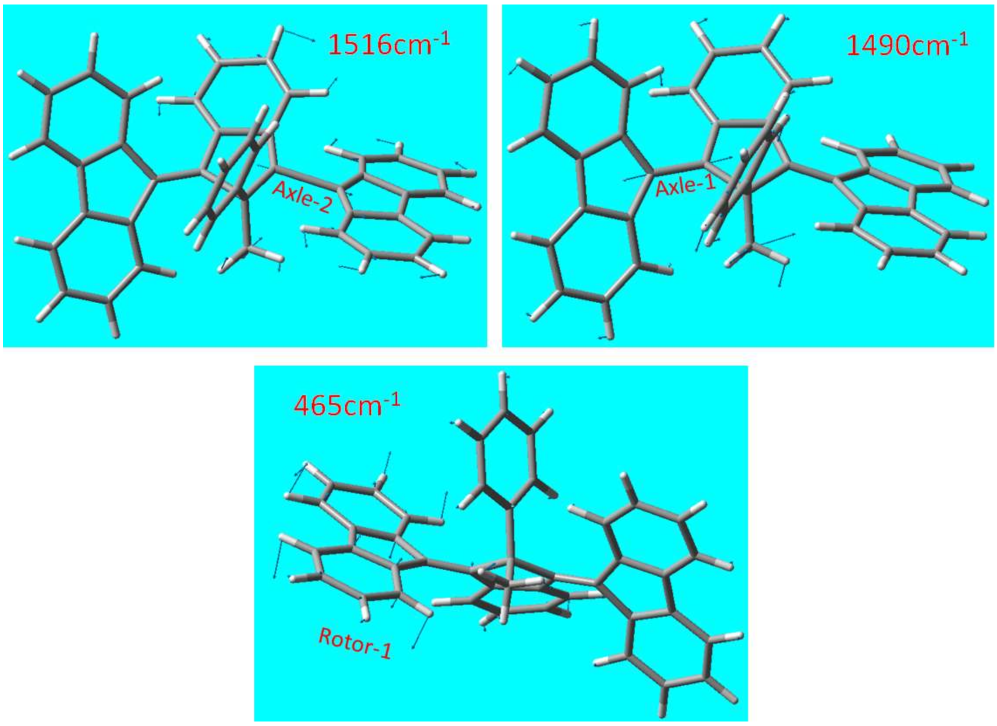

Supplementary Figure 19: 1516, 1490 and 465 cm^{-1} modes obtained from Gaussian DFT calculation of the metastable form. The 1516 and 1490 cm^{-1} modes represent ethylenic C=C stretching of individual axle-2 and axle-1 respectively. The 465 cm^{-1} mode represents C,H out-of-plane bending motion around rotor-1 (rotor-1 is rotating around axle-1).

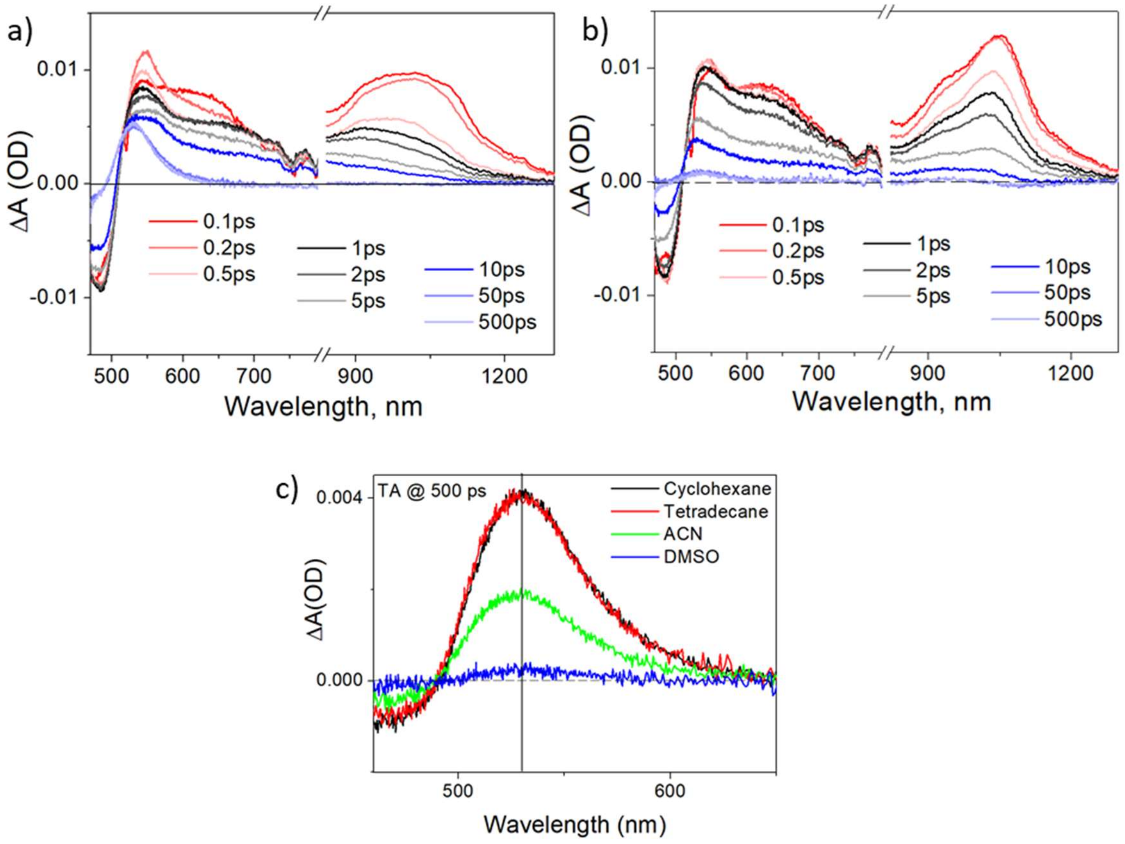

Supplementary Figure 20: Transient absorption spectra of 3GMph in a) tetradecane and b) DMSO are recorded at different pump-probe delays. c) TA spectra at 500 ps are plotted for comparison in solvents with different polarity and viscosity. The measurements were done under similar experimental conditions (sample concentration, pump power and overlap) in order to compare the relative yield of metastable product formation.

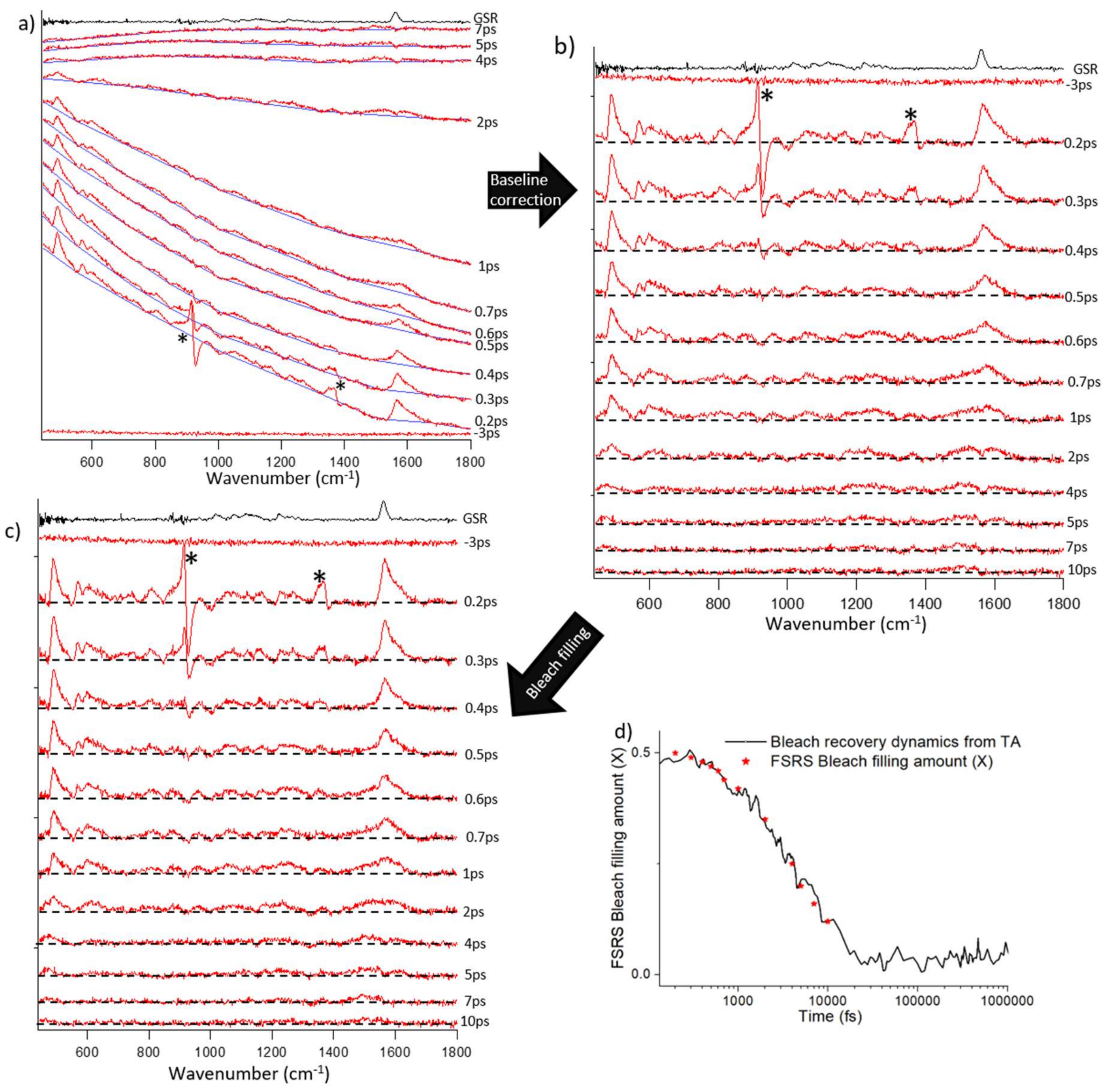

Supplementary Figure 21: a) Raw excited state Raman data (ESR, Raman pump at 700 nm) of 3GMph in ACN at different time delays with the corresponding baselines (blue) are shown. b) The baseline corrected raw ESR spectra are shown. c) The bleach filled ESR spectra at different time delays are shown. Asterisk (*) shows the region of solvent artifact. d) The corresponding bleach filling amount at different time delays are plotted (marked by red star). The bleach filling dynamics from FSRS follows the ground state bleach recovery dynamics from TA (black line).

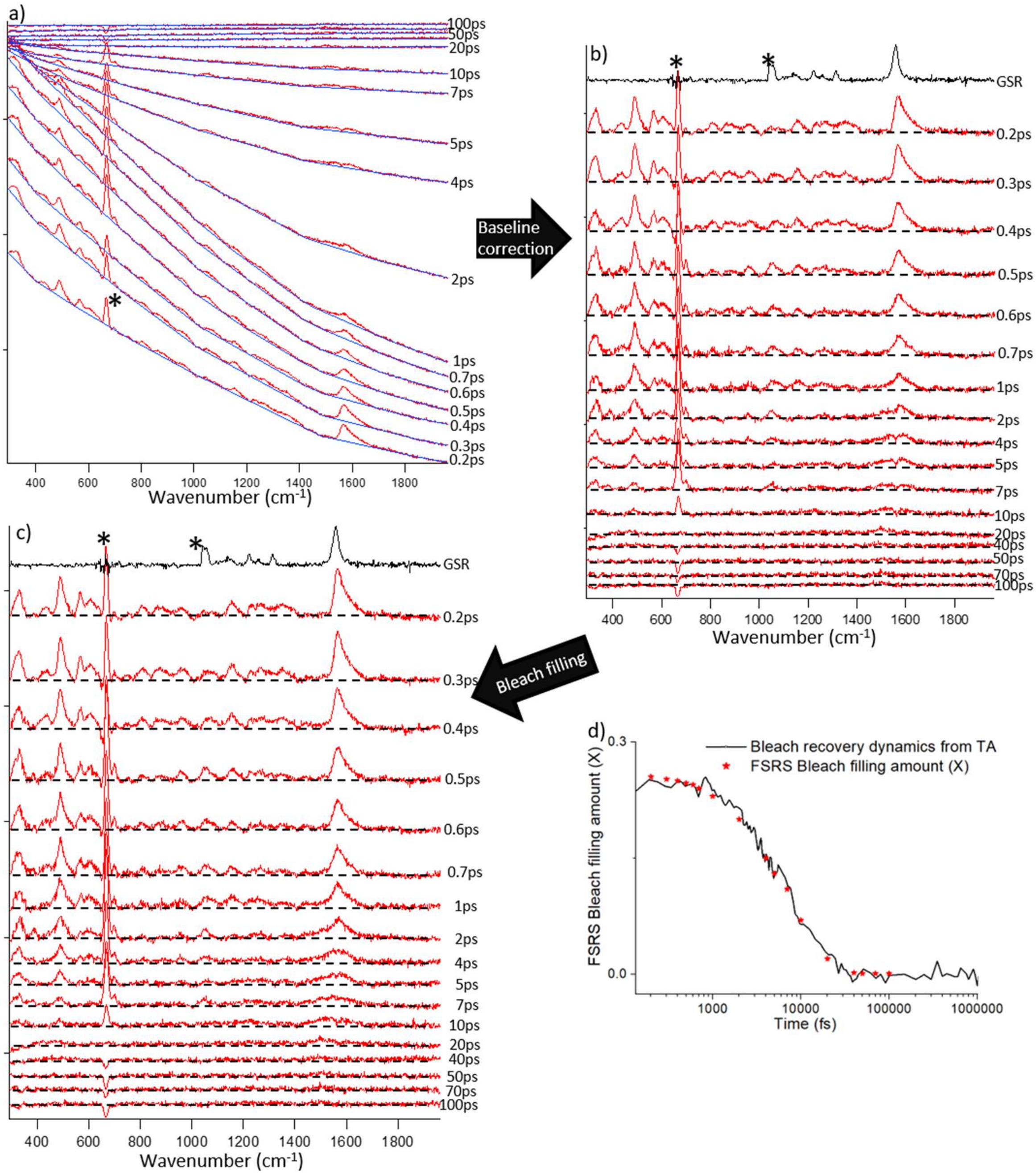

Supplementary Figure 22: a) Raw excited state Raman data (ESR, Raman pump at 650 nm) of 3GMph in DMSO at different time delays with the corresponding baselines (blue) are shown. b) The baseline corrected raw ESR spectra are shown by red color. The black spectrum represents ground state Raman signal. c) The bleach filled ESR spectra at different time delays are shown. Asterisk (*) shows the region of solvent artifact. d) The corresponding bleach filling amount at different time delays are plotted (marked by red star). The bleach filling dynamics from FSRS follows the ground state bleach recovery dynamics from TA (black line).

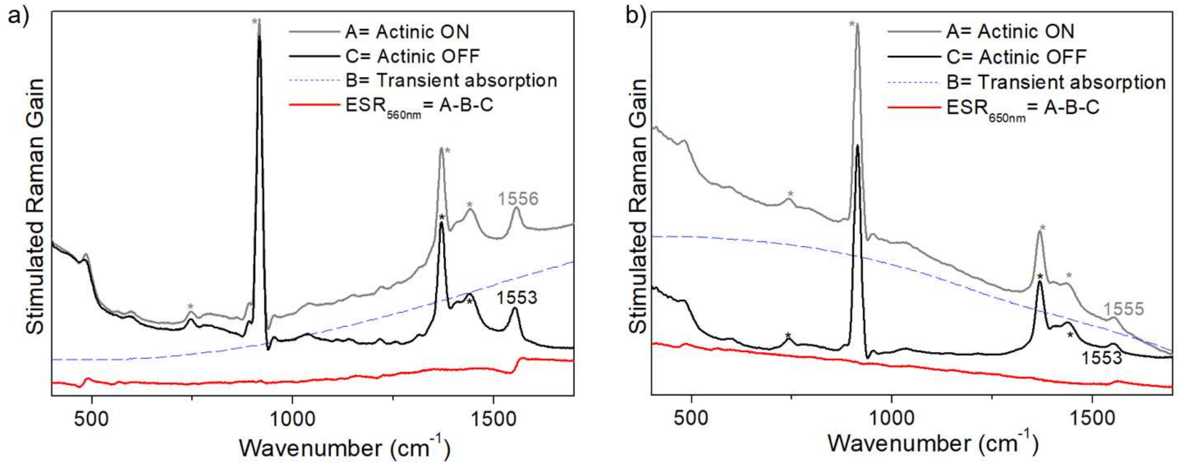

Supplementary Figure 23: Procedure to generate raw excited state Raman data (ESR) from the unprocessed FSRS signal of 3GMph in ACN at 200 fs utilising Raman pump at a) 560 and b) 650 nm. The transient absorption (blue dotted line, B) and ground state Raman (black solid line, C) contributions have been subtracted from its unprocessed FSRS data with actinic ON (grey solid line, A) to generate the ESR_{560} and ESR_{650} (red solid line).

Atomic coordinates of stable 3GMph optimized at rb3lyp/tzvp level of calculation:

C	1.19606400	0.15808500	0.34723800
C	0.00006100	-0.82704800	0.11173200
C	-1.19600900	0.15806400	0.34716500
C	0.70238200	1.10429900	1.34999300
C	-0.70010000	2.45886900	3.33282500
C	-0.70239100	1.10426900	1.34996500
C	-1.40520300	1.77508500	2.35476700
H	-2.48538600	1.73843400	2.38652500
C	2.41979100	0.20448900	-0.25760500
C	3.21837300	-0.86972400	-0.91375000
C	3.26241600	1.43265700	-0.38703900
C	3.02239000	-2.24594700	-1.05188100
C	4.45842200	-0.32706600	-1.33048000
C	2.98303900	2.77893600	-0.14838000
C	4.46861600	1.10036900	-1.03947500
C	4.01161300	-3.03764300	-1.62956500
H	2.11643300	-2.72315100	-0.71786900
C	5.44562000	-1.12055800	-1.90161000
C	3.92326500	3.74996000	-0.47981500
H	2.04400000	3.08711000	0.28534700
C	5.40942100	2.07113500	-1.36682600
C	5.21527700	-2.48270700	-2.05901500
H	3.83901600	-4.10112900	-1.74111100
H	6.38818900	-0.68627800	-2.21269700

C	5.13854300	3.40140900	-1.06970100
H	3.70149700	4.79168000	-0.28223200
H	6.33148200	1.79948600	-1.86675700
H	5.97440100	-3.11387300	-2.50457200
H	5.85948600	4.17064600	-1.31806400
H	-1.23625000	2.96870000	4.12388800
C	-2.41969900	0.20447000	-0.25774100
C	-3.26239700	1.43261700	-0.38703600
C	-3.21829200	-0.86971800	-0.91395200
C	-2.98298100	2.77888900	-0.14840000
C	-4.46868300	1.10032100	-1.03929400
C	-3.02223700	-2.24589900	-1.05239900
C	-4.45844100	-0.32708500	-1.33043500
C	-3.92327200	3.74991300	-0.47965700
H	-2.04387200	3.08704700	0.28518500
C	-5.40955900	2.07108400	-1.36645600
C	-4.01148500	-3.03755700	-1.63009500
H	-2.11619900	-2.72311700	-0.71863000
C	-5.44567100	-1.12053700	-1.90155900
C	-5.13864600	3.40135300	-1.06933900
H	-3.70147900	4.79163500	-0.28211100
H	-6.33171000	1.79945000	-1.86623000
C	-5.21526000	-2.48264000	-2.05925300
H	-3.83880400	-4.10100400	-1.74189000
H	-6.38831200	-0.68625000	-2.21241700

H	-5.85964300	4.17058200	-1.31757200
H	-5.97439900	-3.11378000	-2.50482400
C	0.69993500	2.45891000	3.33285000
H	1.23602100	2.96876400	4.12394100
C	1.40511700	1.77514600	2.35482900
H	2.48529700	1.73851100	2.38665200
C	0.00014400	-1.42896600	-1.31962500
H	0.00014200	-2.51737900	-1.30820900
H	-0.87078700	-1.10310900	-1.87825100
H	0.87116400	-1.10312400	-1.87812800
C	0.00000900	-1.89594000	1.24322400
C	-1.19532400	-2.38491200	1.77914000
C	1.19529900	-2.38452100	1.77960800
C	-1.19746600	-3.35667900	2.77486300
H	-2.14171000	-1.99339800	1.43629100
C	1.19737400	-3.35628000	2.77533000
H	2.14168200	-1.99269400	1.43709800
C	-0.00006300	-3.85539500	3.27340400
H	-2.14220900	-3.71318500	3.16760400
H	2.14207500	-3.71248100	3.16844900
H	-0.00008200	-4.60912900	4.05126600

Atomic coordinates of metastable 3GMph optimized at rb3lyp/tzvp level of calculation:

C	-1.33035400	0.48104700	0.04894300
C	-0.08019200	-0.39504300	0.32764500
C	1.09029900	0.64969500	0.40108700
C	-0.85498500	1.82410100	-0.24467200
C	0.44341300	4.27533200	-0.52944300
C	0.53547700	1.92281400	-0.01218300
C	1.15454800	3.18340200	-0.07100700
H	2.18683400	3.29605900	0.22615500
C	-2.64436200	0.09114500	0.15146700
C	-3.24305400	-1.25353400	-0.06448300
C	-3.80085100	0.96682900	0.50004900
C	-2.69698100	-2.47985500	-0.45062900
C	-4.65257900	-1.14817400	0.03373300
C	-3.85789500	2.24244800	1.06562200
C	-4.99306800	0.21345800	0.42407200
C	-3.53258200	-3.56793200	-0.69054600
H	-1.63480600	-2.60935300	-0.57439000
C	-5.48274700	-2.23267100	-0.21678900
C	-5.08474300	2.78132700	1.44026500
H	-2.96147900	2.82168500	1.22864100
C	-6.22002100	0.75527300	0.79086800
C	-4.91606200	-3.45191200	-0.57416900
H	-3.09411500	-4.51583800	-0.97742600
H	-6.55882500	-2.13128600	-0.14119100

C	-6.26535700	2.05464500	1.28269000
H	-5.11875300	3.77577300	1.86833700
H	-7.12587200	0.16474200	0.72309200
H	-5.54997800	-4.30821100	-0.76953700
H	-7.21250700	2.49177500	1.57424300
H	0.92394100	5.24417500	-0.58905300
C	2.43864100	0.40687900	0.58950500
C	3.11875900	-0.50587800	1.53230200
C	3.52900700	0.95710200	-0.26377100
C	2.68667100	-1.11009000	2.71701200
C	4.50737800	-0.52321100	1.24516700
C	3.49864000	1.71815600	-1.43633900
C	4.75142000	0.34515300	0.10390500
C	3.58725100	-1.81851000	3.50746700
H	1.66999800	-1.00907800	3.05655400
C	5.40505300	-1.22646500	2.03852900
C	4.66860300	1.93406400	-2.15542400
H	2.57653000	2.14338700	-1.80297200
C	5.91966200	0.56063100	-0.61952000
C	4.93598700	-1.89865400	3.16243700
H	3.23541700	-2.29503200	4.41447800
H	6.46251000	-1.23036800	1.80218600
C	5.88006500	1.37538300	-1.74405200
H	4.63410300	2.53957500	-3.05303000
H	6.84526100	0.08053300	-0.32460700

H	5.62368800	-2.45122700	3.79084400
H	6.78162000	1.55596900	-2.31650800
C	-0.88963100	4.13073200	-0.94265000
H	-1.41959000	4.97789400	-1.36042000
C	-1.53982400	2.91989500	-0.79302100
H	-2.57340000	2.82402700	-1.09039600
C	-0.34213600	-1.20601200	1.60967500
H	-1.28115600	-1.74696500	1.53413500
H	0.44074200	-1.92866700	1.80717400
H	-0.41767300	-0.53210800	2.46396800
C	0.30545300	-1.27271400	-0.88993900
C	1.09798800	-2.41495700	-0.74122900
C	-0.07669000	-0.90608400	-2.18327400
C	1.47324500	-3.17494500	-1.84402700
H	1.43790300	-2.72379800	0.23700500
C	0.29990900	-1.66306000	-3.28744600
H	-0.69014600	-0.02779100	-2.33420400
C	1.07476300	-2.80501500	-3.12382500
H	2.08340600	-4.05825600	-1.69838800
H	-0.01915900	-1.35845200	-4.27692300
H	1.36680400	-3.39793400	-3.98178200

Supplementary References

1. Kistemaker, J.C.M., et al., *Third-Generation Light-Driven Symmetric Molecular Motors.* Journal of the American Chemical Society, 2017. **139**(28): p. 9650-9661.
2. Galbán, J., et al., *The intrinsic fluorescence of FAD and its application in analytical chemistry: a review.* Methods Appl Fluoresc, 2016. **4**(4): p. 042005.
3. Heisler, I.A., M. Kondo, and S.R. Meech, *Reactive Dynamics in Confined Liquids: Ultrafast Torsional Dynamics of Auramine O in Nanoconfined Water in Aerosol OT Reverse Micelles.* Journal of Physical Chemistry B, 2009. **113**(6): p. 1623-1631.
4. Hall, C.R., et al., *Ultrafast Dynamics in Light-Driven Molecular Rotary Motors Probed by Femtosecond Stimulated Raman Spectroscopy.* Journal of the American Chemical Society, 2017. **139**(21): p. 7408-7414.
5. Snellenburg, J.J., et al., *Glotaran: A Java-Based Graphical User Interface for the R Package TIMP.* Journal of Statistical Software, 2012. **49**(3): p. 1-22.
6. M. J. Frisch, G.W.T., H. B. Schlegel, G. E. Scuseria, M. A. Robb, J. R. Cheeseman, J. A. Montgomery, Jr., T. Vreven, K. N. Kudin, J. C. Burant, J. M. Millam, S. S. Iyengar, J. Tomasi, V. Barone, B. Mennucci, M. Cossi, G. Scalmani, N. Rega, G. A. Petersson, H. Nakatsuji, M. Hada, M. Ehara, K. Toyota, R. Fukuda, J. Hasegawa, M. Ishida, T. Nakajima, Y. Honda, O. Kitao, H. Nakai, M. Klene, X. Li, J. E. Knox, H. P. Hratchian, J. B. Cross, C. Adamo, J. Jaramillo, R. Gomperts, R. E. Stratmann, O. Yazyev, A. J. Austin, R. Cammi, C. Pomelli, J. W. Ochterski, P. Y. Ayala, K. Morokuma, G. A. Voth, P. Salvador, J. J. Dannenberg, V. G. Zakrzewski, S. Dapprich, A. D. Daniels, M. C. Strain, O. Farkas, D. K. Malick, A. D. Rabuck, K. Raghavachari, J. B. Foresman, J. V. Ortiz, Q. Cui, A. G. Baboul, S. Clifford, J. Cioslowski, B. B. Stefanov, G. Liu, A. Liashenko, P. Piskorz, I. Komaromi, R. L. Martin, D. J. Fox, T. Keith, M. A. Al-Laham, C. Y. Peng, A. Nanayakkara, M. Challacombe, P. M. W. Gill, B. Johnson, W. Chen, M. W. Wong, C. Gonzalez, and J. A. Pople, Gaussian 03, Revision A.1,Gaussian, Inc., Pittsburgh PA,, 2003.
